# Phenology and Productivity of C_3_ and C_4_ Grasslands in Hawaii

**DOI:** 10.1371/journal.pone.0107396

**Published:** 2014-10-07

**Authors:** Stephanie Pau, Christopher J. Still

**Affiliations:** 1 Department of Geography, Florida State University, Tallahassee, Florida, United States of America; 2 Forest Ecosystems and Society, Oregon State University, Corvallis, Oregon, United States of America; Helmholtz Centre for Environmental Research - UFZ, Germany

## Abstract

Grasslands account for a large proportion of global terrestrial productivity and play a critical role in carbon and water cycling. Within grasslands, photosynthetic pathway is an important functional trait yielding different rates of productivity along environmental gradients. Recently, C_3_-C_4_ sorting along spatial environmental gradients has been reassessed by controlling for confounding traits in phylogenetically structured comparisons. C_3_ and C_4_ grasses should sort along temporal environmental gradients as well, resulting in differing phenologies and growing season lengths. Here we use 10 years of satellite data (NDVI) to examine the phenology and greenness (as a proxy for productivity) of C_3_ and C_4_ grass habitats, which reflect differences in both environment and plant physiology. We perform phylogenetically structured comparisons based on 3,595 digitized herbarium collections of 152 grass species across the Hawaiian Islands. Our results show that the clade identity of grasses captures differences in their habitats better than photosynthetic pathway. Growing season length (GSL) and associated productivity (GSP) were not significantly different when considering photosynthetic type alone, but were indeed different when considering photosynthetic type nested within clade. The relationship between GSL and GSP differed most strongly between C_3_ clade habitats, and not between C_3_-C_4_ habitats. Our results suggest that accounting for the interaction between phylogeny and photosynthetic pathway can help improve predictions of productivity, as commonly used C_3_-C_4_ classifications are very broad and appear to mask important diversity in grassland ecosystem functions.

## Introduction

A critically important problem in understanding ecosystem responses to global change is the relationship between growing season length and productivity. This is because growing season length is commonly used to predict net ecosystem exchange and to inform terrestrial biosphere models of vegetation dynamics and carbon exchange [1], [2]. The length of the growing season should be a primary factor controlling the terrestrial carbon cycle by setting the length of time available for photosynthesis and plant growth [3]–[5]. For example, an extension of the growing season either through earlier spring onset or later autumn senescence has been shown to be positively related to primary productivity and increased carbon assimilation across several different vegetation types [6] and refs. above but also see [7]–[8] for examples of no or negative relationships. However most studies examine temperate deciduous or evergreen forests, while examples from grasslands and tropical sites are limited.

Grasslands and tropical savannas comprise approximately 29% of the global extent of terrestrial biomes and this corresponds to some 33% of global terrestrial NPP [9]. Photosynthetic pathway is a dominant plant functional trait that has consequences for the global carbon cycle because of the distribution of C_3_ and C_4_ plants and their different rates of productivity [10]. In general, the efficiency of C_4_ photosynthesis should be greater than C_3_ photosynthesis under conditions of high temperatures, high light, and greater aridity [11], [12]. The classic turnover of C_3_-C_4_ grasses along altitudinal gradients has been well documented [13]–[19]. This turnover illustrates the physiological advantage of C_4_ grasses in warmer and drier environments, which confers dominance in species composition and relative cover. The occurrence of C_4_ grasses in more marginal environments, however, should have consequences for their productivity; thus they may not be more productive than C_3_ grasses even with an advantage in resource-use efficiencies under resource-poor conditions [15].

C_3_ and C_4_ grasses should also partition their activity along temporal environmental gradients resulting in different phenologies and growing season lengths. Differences in phenology have been demonstrated in both mixed C_3_-C_4_ grasslands [20], [21] as well as across large environmental gradients [15]. Studies of C_3_ and C_4_ grasslands in the Great Plains of North America have demonstrated that, compared to C_3_ grasses, C_4_ grasses are active later in the growing season when temperatures warm and water becomes limiting in their respective environment [21]–[25]. The Great Plains region has been the focus of many subsequent studies using remote sensing to discriminate C_3_-C_4_ regions based on their distinct seasonality and responses to climate variability [26]–[29].

Yet there is a growing body of work that has re-examined C_3_-C_4_ comparisons, benefiting from the recent development of well-resolved grass phylogenies [30]. The vast majority of grasses (Poaceae) belong to either the ‘BEP’ or ‘PACMAD’ clade, which last shared a common ancestor an estimated 50–80 Mya [31]. ‘BEP’ is an acronym for the Bambusoideae, Ehrhartoideae, and Pooideae lineages, which contain only C_3_ species. ‘PACMAD’ refers to Panicoideae, Aristidoideae, Chloridoideae, Micrairioideae, Arundinoidae, and Danthonioideae lineages, which contain all of the C_4_ species and some C_3_ species [30]. Thus, there is considerable diversity within C_3_ and C_4_ groups associated with different lineages. Comparing C_3_ and C_4_ grasses restricted to the PACMAD clade enables our understanding of ecological differences previously confounded by different evolutionary histories [32]–[37]. Furthermore C_3_ BEP and PACMAD taxa may exhibit ecological differences relevant to community structure and ecosystem functions.

Previous phylogenetic modeling of Hawaiian grasses has demonstrated that BEP and PACMAD clades capture habitat distinctions not strictly associated with photosynthetic pathway [33]. This work suggested that there are larger differences in aridity between the habitats of C_3_ and C_4_ grasses compared to temperature differences [33], [35], [37], a finding largely driven by the much higher precipitation and woody cover of C_3_ PACMAD grass environments compared to other grass groupings [38]. Subsequent ecological niche modeling described the changes from C_4_ grass-dominated habitats in lower elevation, warm and dry regions, to C_3_ PACMADs in mid-elevation, cooler and wetter regions, and finally to C_3_ BEPs at the highest elevations in cooler and drier conditions [37]. It was furthermore shown that C_3_ PACMADs tend to occur in habitats that receive early season (winter) precipitation, whereas the habitat sorting of C_3_ BEPs and C_4_ grasses was associated with temperature differences. This work and others add to the growing evidence that there are important ecological differences among lineages that were not captured by previous C_3_-C_4_ comparisons. Likewise, phylogenetically structured comparisons should highlight when and where photosynthetic pathway may in fact be responsible for habitat sorting and differences in resource strategies.

In this study we examine the phenology and inferred productivity of C_3_-C_4_ grass habitats using herbarium collection localities across the Hawaiian Islands and a timeseries of satellite images. C_3_ and C_4_ grasses dominate distinct regions along spatial gradients, thus our examination of the phenology of these habitats reflects both the dominant plant functional type and the environment in which they occur. We compare the habitats of C_3_ and C_4_ grass species within the PACMAD clade to try to isolate the effects of photosynthetic pathway on the timing, magnitude, and estimated productivity (integrated NDVI) of these grasslands. We also examine habitats of C_3_ grass species from both PACMAD and BEP clades to highlight the functional diversity that is commonly grouped. Specifically, we ask the following questions: Do the habitats of C_3_ and C_4_ grass species within and between clades differ in their phenology (start-of-season, end-of-season, and growing season length) and productivity? Is the relationship between growing season length and productivity of grass species' habitats within and between clades different? The Hawaiian Islands provide an ideal setting for this work because of the considerable variation in both species diversity and climatic gradients within a small geographic region. The majority of grasses in Hawaii are introduced, and thus their distribution should reflect recent ecological sorting and not insular evolutionary history or patterns of extinction.

## Methods

### Species and environmental data

We used a dataset of 3,595 digitized geo-referenced herbarium records for Poaceae created by [33]. Records from across the main seven Hawaiian Islands (Kauai, Oahu, Molokai, Lanai, Maui, Kahoolawe, and Hawaii) were obtained from the Smithsonian Flora of the Hawaiian Islands Website and The Bishop Museum Herbarium. The dataset represents 152 grass species (there are multiple collections for most species), each assigned to either the ‘BEP’ or ‘PACMAD’ clade that together represent most species of Poaceae. Each species' life history was further categorized as annual or perennial based on [39]. Because individual occurrence records may not indicate a large enough expanse of grasses for remote sensing analysis, herbarium point localities were excluded if they did not fall within a grassland landcover class according to the Hawaii GAP Analysis. The Hawaii GAP Analysis is a land cover dataset derived from 30-m resolution Landsat imagery from 1999–2003 and edited using ancillary data and expert knowledge (US Geological Survey, Gap Analysis Program (GAP) August 2011. National Land Cover, Version 2). As a consequence, our C_3_ PACMAD samples represent species that occur open grassland environments, yet C_3_ PACMADs tend to occur in regions with the greater tree cover [37], [38].

Additionally, C_3_ PACMADs present a challenge in using remotely sensed data to capture grass phenology because they are globally uncommon. Because of this, we verified that at least ten C_3_ PACMAD species in our herbarium records were collected recently based on collection dates. Five species have been collected at recently as 2000 (*Cortaderia jubata*, *Dichanthelium hillebrandianum*, *Oplimensus compositus*, *Rytidosperma pilosum*, and *Schizostachyum glaucifolium*); in addition, another five have been collected since 1990 (*Cortaderia selloana*, *Dichanthelium cynodon*, *Isachne distichophylla*, *Oplimenus hirtella*, *Sacciolepsis indica*). These species are furthermore all considered common and/or occurring densely in open areas in Hawaii, with the exception of *Schizostachum* [39].

### Remote sensing data

We used the NASA Terra Moderate Resolution Imaging Spectroradiometer (MODIS) NDVI (MOD13Q1) Collection 5, which provides 16-day composite data at 250-m spatial resolution. Data were screened using the ‘QC_Day’ scientific data set for only ‘good quality’ pixels (i.e., not contaminated by clouds or aerosols) or ‘check other QA’ in the VI Quality dataset. Pixels falling into the latter category were discarded if flagged for clouds (adjacent cloud, mixed clouds and possible shadow), aerosols (high and climatology aerosols), or possible shadow. NDVI has been shown to be less sensitive to view-angle differences compared to the other commonly used index of plant greenness, the Enhanced Vegetation Index (EVI) [40].

An NDVI time series was created from one pixel centered over each collection locality for a 10-year period using 16-day MODIS data (February 2000–February 2010). Each time series was first smoothed using a centered moving-window average of five data points. For each smoothed time series (for each collection locality), piece-wise logistic functions were fit to each ascending and descending phase of NDVI, following [41]. The points of local maxima or minima in the rate of change in the curvature of the fitted logistic models were used to identify the ‘Start-of-Season’ (SOS) and ‘End-of-Season’ (EOS) for each year [41]. ‘Growing Season Length’ (GSL) is the length of time between SOS and EOS, and ‘Growing Season Productivity’ (GSP) is the time-integrated NDVI between the SOS and EOS (integrated across days - NDVI is a ratio and is unitless). An attempt was made to use each of the 3,595 herbaria point localities for logistic fits. However, in a given year, collection localities were excluded if the fitting procedure failed to converge, for example, because too few data points remained after the QC_Day screening.

On average 107 logistic fits were made each year for C_3_ BEPs, 19 for C_3_ PACMADs, and 284 for C_4_ PACMADs, which in part reflects the number of collections for each group (there were fewer C_3_ PACMADs in the herbarium records) as well as potential biases in tree-covered and cloudy regions that were screened more heavily. Of these successful logistic fits, SOS and EOS dates were then averaged across all species falling into each photosynthetic type-clade combination (3 groups: C_3_ PACMAD, C_3_ BEP, and C_4_ PACMAD), for each year (10 years). Annuals and perennials within each photosynthetic type-clade combination were also determined (except there were no annual C_3_ PACMADS), which together with the 3 photosynthetic type-clade combinations produced a total of 50 samples for statistical analyses.

### Statistical analyses

First, linear models were used to examine the effect of photosynthetic pathway on SOS, EOS, and GSL irrespective of clade (all C_3_ vs. C_4_ grasses). Then, photosynthetic pathway nested within clades (PACMAD:C_3_, PACMAD:C_4_, BEP:C_3_) were used to assess differences in SOS, EOS, and GSL. Using photosynthetic type nested within clade tests for differences in means among photosynthetic type and differences in means among photosynthetic type-clade combinations. Tukey post-hoc tests were used to examine independent contrasts between each group and to test for differences more likely due to photosynthetic type by controlling for clade identity (C_3_ PACMAD vs. C_4_ PACMAD). Linear models were also used to examine the relationship between GSL and GSP. Four models were compared using a corrected Akaike's Information Criterion (AICc) for small sample sizes and with greater penalties for extra model parameters [42]. The simplest model considered only GSL as a predictor of GSP (Model A; see Table 1). Then the interactions between GSP and clade (Model B), between GSP and photosynthetic type (Model C), and between GSP and photosynthetic pathway nested within clade (Model D) were included. These models were further compared using Akaike weights (*w*_i_), which indicate a measure of model selection uncertainty, i.e., the probability that a model is ‘best’ among a set of candidate models.

**Table 1:** Comparison of models predicting growing season productivity (GSP; estimated with integrated NDVI) using growing season length (GSL) and its interaction with clade and photosynthetic type.

Model parameters	ΔAICc	Akaike weights (*w_i_*)	k
Model A: GSL	23.823	<0.001	3
Model B: GSL*clade	**1.770**	**0.292**	**5**
Model C: GSL*photosynthetic type	25.003	<0.001	5
Model D: GSL*clade:photosynthetic type	**0**	**0.708**	**5**

Equivalent best models (when ΔAICc is less than or equal to 2) are highlighted in bold and show the importance of clade identity. ‘k’ = number of model parameters.

## Results and Discussion

**Figure 1:**
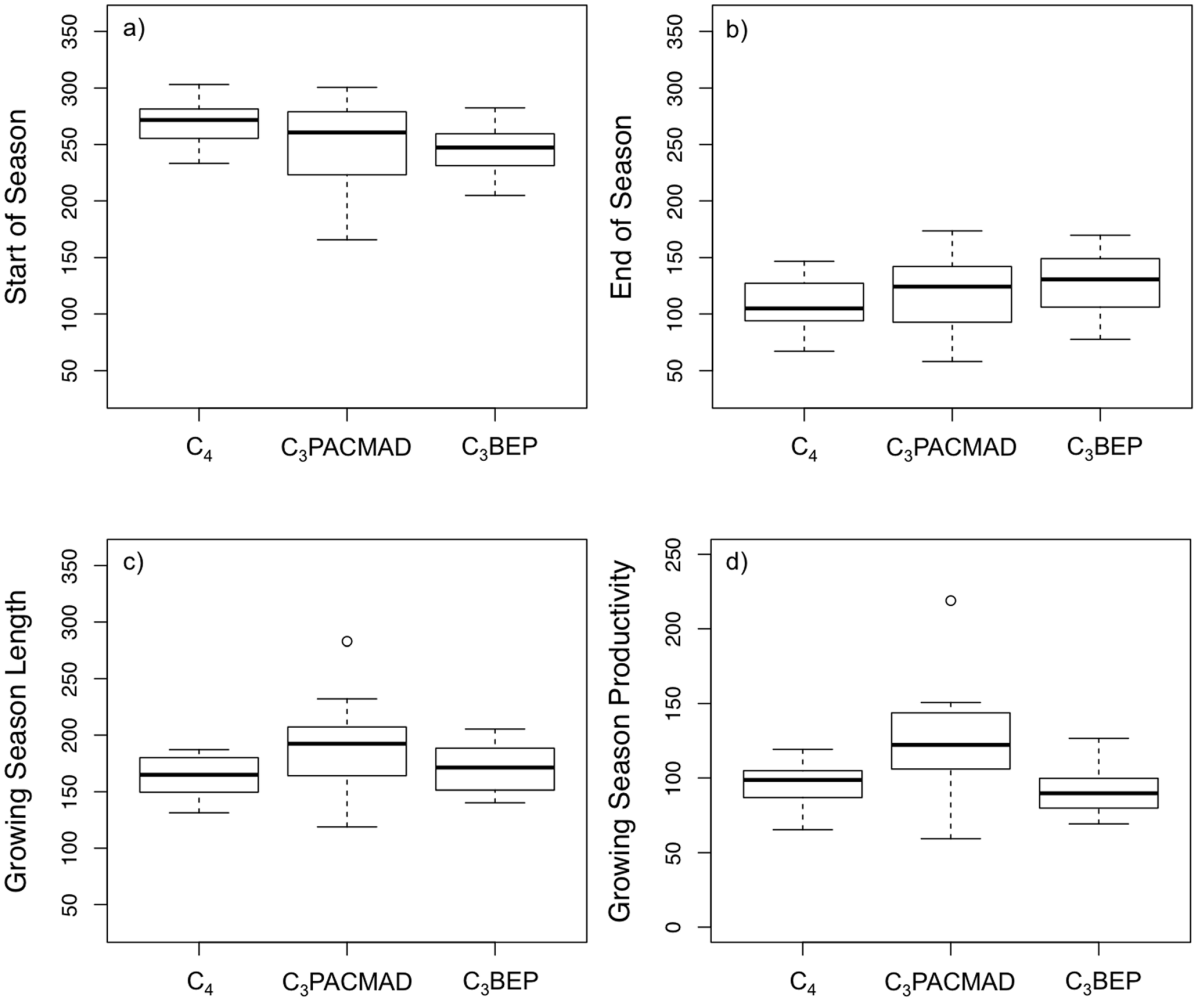
Differences in C_3_ and C_4_ start-of-season (SOS) (a), end-of-season (EOS) (b), growing season length (c), and growing season productivity (d). Y-axis for panels (a)–(c) is the Julian day-of-year or DOY; y-axis for panel (d) is integrated NDVI, which is unitless, based on logistic models using 10-year timeseries of MODIS NDVI (see Methods). The growing season in Hawaii crosses the calendar year so that SOS begins at a later DOY than EOS. Most Poaceae species fall within the ‘BEP’ or ‘PACMAD’ clade. All C_4_ grasses are in the ‘PACMAD’ clade.

Ignoring phylogeny (i.e., comparing only C_3_ and C_4_ grass groupings), SOS was later in C_4_ habitats compared to C_3_ habitats (F = 9.42, p<0.01, df = 48; Figure 1a). The SOS for C_4_ habitats occurred during the end of September (mean day of year or DOY = 269 based on logistic models) compared to the beginning of September for C_3_ habitats (mean DOY = 245). In Hawaii, climate seasonality is governed primarily by precipitation, with November-April generally considered the wet season and May-October the dry season [39], [43]. Therefore, the SOS for both C_3_ and C_4_ grasslands appear to fall within the dry season, but C_4_ habitats green-up later in the dry season when plants should be more drought-stressed. C_4_ grasses in temperate climates have been shown to be active and more productive later in the growing season as temperatures increase and water becomes limiting in their respective environments [20], [25], [44]. Indeed, this temporal offset forms the basis for predicting C_3_-C_4_ mixtures in temperate grasslands [26]–[29], [45], [46]. The physiological advantage of greater water-use efficiency [47] and dominance of arid, warm environments (reviewed in [12]) by C_4_ grasses is well recognized and furthermore shown to be robust in phylogenetically structured screening experiments [36].

When we considered phylogenetic structure by accounting for photosynthetic pathway nested within clade, results revealed that the temporal offset in SOS between photosynthetic types reflected differences between C_4_ PACMAD habitats and C_3_ BEP habitats (F = 4.66, p<0.02, df = 47; posthoc Tukey's p<0.05; Figure 1a). C_4_ habitats had a significantly later SOS compared to C_3_ BEP habitats by about 23 days on average (p = 0.03). There was no statistically significant SOS difference between C_3_ and C_4_ PACMADs (posthoc Tukey's p = 0.17). Thus SOS differences were due to BEP-PACMAD identity rather than strictly photosynthetic pathway, otherwise this difference should be apparent in the C_3_ and C_4_ PACMAD comparison. Our data show large variability in SOS for C_3_ PACMAD habitats indicating a diversity of strategies and growing environments for this group. Statistical significance notwithstanding, C_4_ habitats started their growing season an estimated 20 days later into the dry season on average compared to C_3_ PACMAD habitats. C_3_ PACMAD SOS was only about 3 days later than C_3_ BEPs on average, and this difference was not statistically significant (p = 0.99).

EOS showed the greatest variability among phenological metrics. Although there was a significant difference when considering photosynthetic type alone (F = 4.85, p<0.05, df = 48), there was no significant difference when photosynthetic type was nested within clade (F = 2.81, p = 0.07, df = 47; post-hoc Tukey's p>0.05 for all comparisons; Figure 1b). C_3_ and C_4_ habitats brown-down during the transition into the dry season in mid-April/beginning of May (mean EOS DOY for C_3_ = 124; mean EOS DOY for C_4_ = 107). C_4_ habitats had an earlier EOS compared to C_3_ habitats when clade identity was not considered, and this difference appears to be due to the later EOS for BEP taxa (Figure 1b). Environmental controls on EOS or leaf senescence are not well understood (Menzel et al. 2006; Taylor et al. 2007). In other analyses of phenological shifts associated with climate change, EOS dates have exhibited more variability in comparison to SOS dates, [48]–[52].

GSL was not significantly different when considering photosynthetic type alone (F = 3.18, p = 0.08, df = 48), but was indeed different when considering photosynthetic type nested within clade (F = 4.44, p<0.02, df = 47; Figure 1c). C_3_ PACMAD habitats had a longer GSL by about 30 days on average compared to C_4_ habitats in the same clade (posthoc Tukey's p<0.05); no other comparisons were significantly different. Thus the data show that physiological and/or habitat differences governing GSL are likely masked when C_3_ taxa are grouped across clades. But when clades are accounted for, the difference in GSL due to photosynthetic pathway becomes more apparent (i.e., when comparing C_3_ and C_4_ PACMAD GSL – Figure 1c). Similarly, GSP was not significantly different with respect to just photosynthetic type (F = 0.10, p = 0.32, df = 48), but was significantly different when considering photosynthetic type nested within clade (F = 9.59, P<0.001, df = 47). C_3_ PACMAD habitats were significantly more productive than C_3_ BEP and C_4_ habitats (post-hoc Tukey's p<0.001 for both comparisons; Figure 1d). The longer GSL and greater GSP of C_3_ PACMAD habitats reflect the difference in their growing environment—they prefer or are possibly restricted to more favorable habitats for plant growth—wetter than C_4_ habitats and warmer than C_3_ BEP habitats [37].

GSL explained a large proportion of the variability in GSP—as GSL increased, there was a corresponding increase in GSP (Figure 1c,d). There were two equivalent best-fit models predicting yearly differences in GSP and these were the two models that included clade as a model parameter (Models B and D). Akaike weights showed more support for the model with photosynthetic type nested within clade (Model D) over the model with clade alone (Model B), and there was strong support for both models over other candidate models that excluded clade. Using photosynthetic type alone to predict GSP resulted in the worst-fit model.

**Figure 2:**
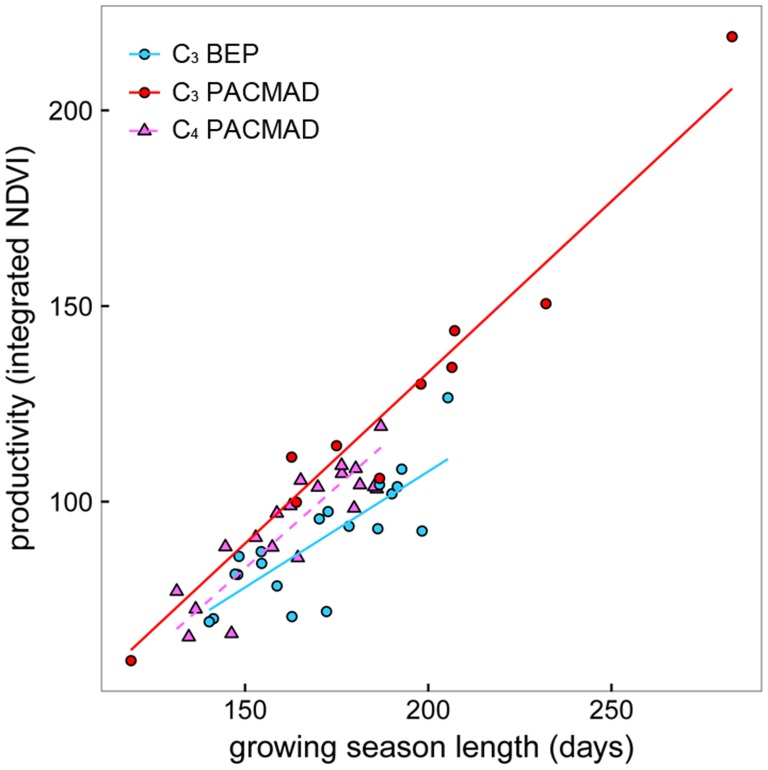
The slope of the relationship between GSL and GSP differed for each photosynthetic type-clade combination (Model D, Table 2). C_3_ PACMAD habitats exhibited higher rates of greenness than C_4_ PACMAD or C_3_ BEP habitats for a given growing season length (slope coefficients = 0.87, 0.83, and 0.59 respectively). Differences within the C_3_ functional group were larger than between photosynthetic pathways, i.e., there was a larger clade effect than photosynthetic pathway.

The slope of the relationship between GSL and GSP differed for each photosynthetic type-clade combination (Model D; F = 108.4, p<0.01, df = 45; Figure 2 and Table 2). Productivity increased more per unit of increase in GSL in C_3_ PACMAD habitats than other grass habitats. Results show that controlling for GSL, C_3_ PACMAD habitats were still more productive than C_4_ or C_3_ BEP habitats. Interestingly, there was a larger difference within the C_3_ functional group than between photosynthetic pathways, i.e., there was a larger clade effect (BEP vs. PACMAD) than photosynthetic pathway effect (C_3_ vs. C_4_). There was not a significant difference in the GSL of habitats where C_3_ BEPs and C_3_ PACMADs occur (Figure 1c), but these habitats had the largest difference in GSP. In comparison to C_4_ regions, BEP environments had a longer GSL but were less productive (Figure 2), although these differences were not statistically significant. This pattern is consistent with a resource strategy of longer growing seasons at a cost of lower productivity [53], [54]. Particularly in resource-poor environments, such as the cold and dry habitats of BEP taxa, species with longer growing seasons are often associated with lower rates of productivity as a strategy to balance energy requirements for tissue growth. BEPs may have traits that have adapted to these marginal environments by adopting a slow and conservative approach to energy-use. Conversely, plants in high-resource environments tend to produce short-lived leaves with high photosynthetic rates at a cost of being less resistant to environmental stresses and having to produce new leaf tissue [54]. This tradeoff between leaf lifespan and rates of photosynthesis has also been demonstrated in tropical species [55], where the growing season is potentially year-round. Although these strategies may explain C_3_ BEP and C_4_ differences, C_3_ PACMADs did not appear to fit this tradeoff, yet they exhibited greater rates of productivity even when controlling for growing season length.

**Table 2:** The relationship between growing season length (GSL) and productivity (GSP) (Model D in Table 1) was marginally different between C_3_ PACMAD and C_4_ grasses (GSL*clade:photosynthetic type), and significantly different between clades irrespective of photosynthetic type (GSL*clade). See Figure 2.

Coefficients	Estimate	Std. Error	t-value	p-value
intercept	−10.80	16.16	−0.67	0.507
GSL	0.59	0.09	6.28	<0.001
clade	−31.11	19.12	−1.63	0.111
GSL*clade	0.28	0.11	2.62	0.012
GSL*clade:photosynthetic type	−0.04	0.02	−2.02	0.049

Notably, life history differences (annual vs. perennial) were not significant in explaining any phenological metric except for GSP (F = 4.63, p<0.05, df = 48), with perennial species being more productive based on integrated NDVI compared to annual species (posthoc Tukey's p<0.05). However the slope of the relationship between GSL and GSP was not significantly different when considering life history (p = 0.07).

Our proxy for productivity (integrated NDVI) only considers aboveground greenness. It is possible that C_4_ and C_3_ BEP grasses may be less productive aboveground because they are allocating resources to roots, a strategy that would make sense in water- or nutrient-limited environments [56]. A further limitation of the study is the influence of tree cover on the phenology of understory grasses. In our case, although we only used pixels occurring in grassland vegetation classes, C_3_ PACMADs preferentially occur in regions with the greater tree cover, which represents a microclimate that is cooler and shadier [37], [38]—consequently these samples were excluded and our C_3_ PACMADs are represented by species that occur only in open environments.

## Conclusions

Our results provide a working hypothesis for understanding C_3_ and C_4_ grass habitats and growing environments. We show that the clade identity of grasses captures differences in their habitats better than photosynthetic pathway. SOS differences that appear to be associated with the habitats of C_3_ and C_4_ grasses are in fact associated with habitats of different clades. EOS was not significantly different among any comparisons. GSL and GSP are indeed constrained by photosynthetic pathway; however, the relationships between GSL and GSP were not as expected given the well-recognized habitat sorting of C_3_-C_4_ photosynthetic pathways. Importantly, the relationship between growing season length and associated productivity differed most strongly between C_3_ clade habitats and not between C_3_-C_4_ habitats. C_3_ PACMAD habitats, which in Hawaii represent highly favorable growing environments, had the longest growing season and exhibited the largest variability in all phenological metrics. Taken together, the characteristics of C_3_ PACMAD habitats shown here may possibly indicate greater trait diversity within this lineage of globally rare grasses and an advantage in adapting to novel climates [57]. Our results highlight the large functional diversity within Poaceae, one of the largest flowering plant families, and how grass species may respond to future global change. We furthermore identify a need for ecosystem and vegetation models of plant productivity to refine relationships between GSL and GSP. Although our work is limited to grasses and their habitats, in theory, different species or plant functional groups should possess distinct growth strategies resulting in complex relationships between phenology and productivity [58].
